# Supplementary material for: A Bacillus subtilis Strain ZJ20 with AFB1 Detoxification Ability: A Comprehensive Analysis
Source: Biology (Basel). 2023 Aug 31;12(9):1195. doi: 10.3390/biology12091195 (PMC10525747; doi:10.3390/biology12091195)
Supplement: Supplementary file 1 [file biology-12-01195-s001.zip › biology-2558127-supplementary.pdf]

Supplementary Material

Table S1. KEGG annotation results

Pathway_ID	Pathway_name	Group	Gene number
ko00471	D-Glutamine and D-glutamate metabolism	Metabolism	5
ko00030	Pentose phosphate pathway	Metabolism	30
ko00564	Glycerophospholipid metabolism	Metabolism	19
ko00680	Methane metabolism	Metabolism	28
ko00040	Pentose and glucuronate interconversions	Metabolism	28
ko04724	Glutamatergic synapse	Organismal Systems	3
ko00270	Cysteine and methionine metabolism	Metabolism	44
ko05120	Epithelial cell signaling in Helicobacter pylori infection	Human Diseases	3
ko00360	Phenylalanine metabolism	Metabolism	17
ko00910	Nitrogen metabolism	Metabolism	22
ko00909	Sesquiterpenoid and triterpenoid biosynthesis	Metabolism	1
ko00642	Ethylbenzene degradation	Metabolism	1
ko03430	Mismatch repair	Genetic Information Processing	26
ko00130	Ubiquinone and other terpenoid-quinone biosynthesis	Metabolism	16
ko05010	Alzheimer's disease	Human Diseases	2
ko00730	Thiamine metabolism	Metabolism	17
ko05111	Vibrio cholerae pathogenic cycle	Human Diseases	9
ko03450	Non-homologous end-joining	Genetic Information Processing	2
ko00260	Glycine	Metabolism	39
ko03440	Homologous recombination	Genetic Information Processing	26
ko04112	Cell cycle - Caulobacter	Cellular Processes	4
ko00620	Pyruvate metabolism	Metabolism	56
ko00473	D-Alanine metabolism	Metabolism	16
ko00010	Glycolysis / Gluconeogenesis	Metabolism	47
ko00592	alpha-Linolenic acid metabolism	Metabolism	1
ko00770	Pantothenate and CoA biosynthesis	Metabolism	19
ko05340	Primary immunodeficiency	Human Diseases	1
ko00430	Taurine and hypotaurine metabolism	Metabolism	8
ko00900	Terpenoid backbone biosynthesis	Metabolism	15
ko00626	Naphthalene degradation	Metabolism	4
ko00280	Valine	Metabolism	35
ko03020	RNA polymerase	Genetic Information Processing	5
ko00903	Limonene and pinene degradation	Metabolism	6
ko00020	Citrate cycle (TCA cycle)	Metabolism	30
ko03410	Base excision repair	Genetic Information Processing	15
ko00523	Polyketide sugar unit biosynthesis	Metabolism	5
ko00061	Fatty acid biosynthesis	Metabolism	28
ko00195	Photosynthesis	Metabolism	8
ko01200	Carbon metabolism	Metabolism	116
ko00071	Fatty acid degradation	Metabolism	26
ko01040	Biosynthesis of unsaturated fatty acids	Metabolism	16
ko00253	Tetracycline biosynthesis	Metabolism	6
ko00120	Primary bile acid biosynthesis	Metabolism	1
ko04066	HIF-1 signaling pathway	Environmental Information Processing	7
ko00312	beta-Lactam resistance	Human Diseases	19

ko00361	Chlorocyclohexane and chlorobenzene degradation	Metabolism	4
ko01053	Biosynthesis of siderophore group nonribosomal peptides	Metabolism	6
ko00780	Biotin metabolism	Metabolism	32
ko04146	Peroxisome	Cellular Processes	10
ko00591	Linoleic acid metabolism	Metabolism	2
ko00906	Carotenoid biosynthesis	Metabolism	1
ko04011	MAPK signaling pathway - yeast	Environmental Information Processing	5
ko04727	GABAergic synapse	Organismal Systems	3
ko00640	Propanoate metabolism	Metabolism	29
ko00980	Metabolism of xenobiotics by cytochrome P450	Metabolism	4
ko03070	Bacterial secretion system	Environmental Information Processing	22
ko01230	Biosynthesis of amino acids	Metabolism	136
ko04726	Serotonergic synapse	Organismal Systems	1
ko04940	Type I diabetes mellitus	Human Diseases	1
ko00720	Carbon fixation pathways in prokaryotes	Metabolism	32
ko00830	Retinol metabolism	Metabolism	4
ko00340	Histidine metabolism	Metabolism	19
ko05134	Legionellosis	Human Diseases	4
ko05203	Viral carcinogenesis	Human Diseases	1
ko00500	Starch and sucrose metabolism	Metabolism	47
ko00950	Isoquinoline alkaloid biosynthesis	Metabolism	3
ko03030	DNA replication	Genetic Information Processing	23
ko00627	Aminobenzoate degradation	Metabolism	13
ko00960	Tropane	Metabolism	3
ko02010	ABC transporters	Environmental Information Processing	146
ko03018	RNA degradation	Genetic Information Processing	21
ko00945	Stilbenoid	Metabolism	1
ko00310	Lysine degradation	Metabolism	14
ko00290	Valine	Metabolism	13
ko00550	Peptidoglycan biosynthesis	Metabolism	26
ko01054	Nonribosomal peptide structures	Metabolism	14
ko04626	Plant-pathogen interaction	Organismal Systems	3
ko00750	Vitamin B6 metabolism	Metabolism	7
ko01051	Biosynthesis of ansamycins	Metabolism	1
ko00072	Synthesis and degradation of ketone bodies	Metabolism	7
ko04622	RIG-I-like receptor signaling pathway	Organismal Systems	1
ko00480	Glutathione metabolism	Metabolism	10
ko04068	FoxO signaling pathway	Environmental Information Processing	3
ko00790	Folate biosynthesis	Metabolism	23
ko00670	One carbon pool by folate	Metabolism	18
ko00300	Lysine biosynthesis	Metabolism	27
ko00940	Phenylpropanoid biosynthesis	Metabolism	1
ko00051	Fructose and mannose metabolism	Metabolism	27
ko00633	Nitrotoluene degradation	Metabolism	1
ko00362	Benzoate degradation	Metabolism	9
ko00230	Purine metabolism	Metabolism	89
ko05152	Tuberculosis	Human Diseases	6
ko00660	C5-Branched dibasic acid metabolism	Metabolism	9

ko00590	Arachidonic acid metabolism	Metabolism	3
ko00450	Selenocompound metabolism	Metabolism	25
ko02060	Phosphotransferase system (PTS)	Environmental Information Processing	27
ko00920	Sulfur metabolism	Metabolism	28
ko05034	Alcoholism	Human Diseases	1
ko02020	Two-component system	Environmental Information Processing	197
ko03420	Nucleotide excision repair	Genetic Information Processing	15
ko00363	Bisphenol degradation	Metabolism	2
ko05014	Amyotrophic lateral sclerosis (ALS)	Human Diseases	3
ko04728	Dopaminergic synapse	Organismal Systems	1
ko00281	Geraniol degradation	Metabolism	2
ko00983	Drug metabolism - other enzymes	Metabolism	9
ko04964	Proximal tubule bicarbonate reclamation	Organismal Systems	2
ko05200	Pathways in cancer	Human Diseases	2
ko00740	Riboflavin metabolism	Metabolism	7
ko01212	Fatty acid metabolism	Metabolism	46
ko00710	Carbon fixation in photosynthetic organisms	Metabolism	12
ko00052	Galactose metabolism	Metabolism	21
ko00524	Butirosin and neomycin biosynthesis	Metabolism	2
ko00970	Aminoacyl-tRNA biosynthesis	Genetic Information Processing	29
ko00520	Amino sugar and nucleotide sugar metabolism	Metabolism	66
ko05146	Amoebiasis	Human Diseases	1
ko00400	Phenylalanine	Metabolism	21
ko01220	Degradation of aromatic compounds	Metabolism	15
ko00240	Pyrimidine metabolism	Metabolism	67
ko03060	Protein export	Genetic Information Processing	22
ko00440	Phosphonate and phosphinate metabolism	Metabolism	1
ko04973	Carbohydrate digestion and absorption	Organismal Systems	1
ko00121	Secondary bile acid biosynthesis	Metabolism	1
ko00250	Alanine	Metabolism	35
ko03050	Proteasome	Genetic Information Processing	2
ko00561	Glycerolipid metabolism	Metabolism	16
ko00630	Glyoxylate and dicarboxylate metabolism	Metabolism	33
ko00600	Sphingolipid metabolism	Metabolism	2
ko01055	Biosynthesis of vancomycin group antibiotics	Metabolism	2
ko00540	Lipopolysaccharide biosynthesis	Metabolism	3
ko04070	Phosphatidylinositol signaling system	Environmental Information Processing	2
ko05132	Salmonella infection	Human Diseases	2
ko05030	Cocaine addiction	Human Diseases	1
ko04910	Insulin signaling pathway	Organismal Systems	1
ko00650	Butanoate metabolism	Metabolism	32
ko00982	Drug metabolism - cytochrome P450	Metabolism	5
ko00401	Novobiocin biosynthesis	Metabolism	4
ko02030	Bacterial chemotaxis	Cellular Processes	36
ko00521	Streptomycin biosynthesis	Metabolism	12
ko00621	Dioxin degradation	Metabolism	3
ko00410	beta-Alanine metabolism	Metabolism	9
ko05031	Amphetamine addiction	Human Diseases	1
ko00232	Caffeine metabolism	Metabolism	1
ko00350	Tyrosine metabolism	Metabolism	11
ko00190	Oxidative phosphorylation	Metabolism	39
ko03320	PPAR signaling pathway	Organismal Systems	6

ko00622	Xylene degradation	Metabolism	3
ko04917	Prolactin signaling pathway	Organismal Systems	1
ko00603	Glycosphingolipid biosynthesis - globo series	Metabolism	1
ko00053	Ascorbate and aldarate metabolism	Metabolism	11
ko00330	Arginine and proline metabolism	Metabolism	50
ko02040	Flagellar assembly	Cellular Processes	34
ko04930	Type II diabetes mellitus	Human Diseases	1
ko00625	Chloroalkane and chloroalkene degradation	Metabolism	15
ko00791	Atrazine degradation	Metabolism	3
ko00760	Nicotinate and nicotinamide metabolism	Metabolism	17
ko01210	2-Oxocarboxylic acid metabolism	Metabolism	34
ko00380	Tryptophan metabolism	Metabolism	14
ko00941	Flavonoid biosynthesis	Metabolism	1
ko05206	MicroRNAs in cancer	Human Diseases	3
ko00472	D-Arginine and D-ornithine metabolism	Metabolism	1
ko03010	Ribosome	Genetic Information Processing	59
ko05150	Staphylococcus aureus infection	Human Diseases	7
ko05204	Chemical carcinogenesis	Human Diseases	1
ko04918	Thyroid hormone synthesis	Organismal Systems	1
ko05211	Renal cell carcinoma	Human Diseases	2
ko00984	Steroid degradation	Metabolism	1
ko00562	Inositol phosphate metabolism	Metabolism	15
ko00785	Lipoic acid metabolism	Metabolism	3
ko04122	Sulfur relay system	Genetic Information Processing	13
ko00311	Penicillin and cephalosporin biosynthesis	Metabolism	5
ko00860	Porphyrin and chlorophyll metabolism	Metabolism	23
ko00460	Cyanoamino acid metabolism	Metabolism	7
ko04920	Adipocytokine signaling pathway	Organismal Systems	5
ko00511	Other glycan degradation	Metabolism	3

Table S2. CAZy database annotation result

gene family	Gene number	Gene ID
		**glycoside hy-drolases(GH)**
GH32	2	NODE_11_88、NODE_2_178
GH23	5	NODE_12_25、NODE_1_620、NODE_10_13、NODE_6_193、NODE_1_501
GH18	2	NODE_1_382、NODE_2_223
GH65	1	NODE_2_169
GH13	4	NODE_2_170、NODE_2_510、NODE_4_96、NODE_9_111
GH68	1	NODE_2_179
GH42	2	NODE_2_210、NODE_4_167
GH53	1	NODE_2_211
GH73	3	NODE_2_42、NODE_2_48、NODE_2_602
GH46	1	NODE_3_484
GH4	4	NODE_4_162、NODE_4_52、NODE_5_214、NODE_7_279
GH42	1	NODE_4_167
GH105	2	NODE_4_175、NODE_5_197
GH51	2	NODE_5_47、NODE_5_69
GH43	2	NODE_5_78、NODE_7_187
GH11	1	NODE_6_149
GH30	1	NODE_6_77
GH1	3	NODE_7_199、NODE_8_7、NODE_9_172
CE12	1	NODE_7_225
GH16	1	NODE_7_230
GH126	1	NODE_8_151
GH26	1	NODE_8_3
GH3	1	NODE_9_10
GH171	1	NODE_9_9
		**glycosyl trans-ferases(GT)**
GT8	1	NODE_11_128
GT51	4	NODE_11_37、NODE_1_764、NODE_2_487、NODE_3_73
GT2	17	NODE_11_78、NODE_11_85、NODE_1_425、NODE_1_478、NODE_2_191、NODE_2_194、NODE_2_196、NODE_2_46、NODE_2_54、NODE_2_55、NODE_2_69、NODE_4_145、NODE_4_16、NODE_6_264、NODE_6_4、NODE_6_5、NODE_8_148
GT28	3	NODE_1_234、NODE_1_429、NODE_3_32
GT83	1	NODE_1_479
GT1	3	NODE_1_551、NODE_6_224、NODE_8_19
GT4	10	NODE_2_190、NODE_2_192、NODE_2_44、NODE_2_65、NODE_3_336、NODE_3_87、NODE_5_272、NODE_5_275、NODE_2_43、NODE_2_70
GT26	1	NODE_2_51
GT35	1	NODE_5_278
GT5	1	NODE_5_279
		**carbohydrate esterases(CE)**
CE4	6	NODE_14_74、NODE_1_564、NODE_1_807、NODE_1_82、NODE_8_62、NODE_4_79
CE9	1	NODE_2_124

CE14	1	NODE_3_88
CE12	2	NODE_4_168、NODE_4_173
CE7	1	NODE_9_145
	**Polysaccharide lyase(PL)**	
PL9	1	NODE_13_26
PL3	1	NODE_2_130
PL1	2	NODE_4_120、NODE_6_129
PL26	1	NODE_4_166
PL11	2	NODE_4_169、NODE_4_170
	**carbohydrate-binding modules(CBM)**	
CBM50	10	NODE_1_487、NODE_1_500、NODE_1_830、NODE_1_835、NODE_3_585、NODE_1_384、NODE_6_202、NODE_6_223、NODE_1_526、NODE_5_8
CBM63	1	NODE_6_127
	**auxiliary activities(AA)**	
AA6	1	NODE_1_870
AA4	1	NODE_5_65
	**COMPOSITE TYPE**	
CBM50+GH18	2	NODE_16_9、NODE_8_20
CBM34+GH13	1	NODE_2_164
CBM66+GH32	1	NODE_3_499
CBM48+CBM68+GH13	1	NODE_5_179
CBM48+GH13	1	NODE_5_282
CBM91+GH43	1	NODE_6_15
CBM3+GH5	1	NODE_6_75
CBM6+GH43	1	NODE_6_78
CBM16+CBM22	1	NODE_7_202
CBM26+GH13	1	NODE_9_131

Table S3. Prediction of secondary metabolites of *B. subtilis* ZJ20

Region	Type	Most similar known cluster	similarity
Region 1.1	lanthipeptide-class-i		
Region 1.2	terpene		
Region 3.1	sactipeptide	subtilosin A	100%
Region 3.2	other	bacilysin	100%
Region 11.1	NRPS		
Region 12.1	CDPS	Pulcherriminic acid	100%
Region 12.2	lanthipeptide-class-i	subtilin	100%
Region 12.3	NRP-metallophore,NRPS	bacillibactin	100%
Region 20.1	T3PKS	1-carbapen-2-em-3-carboxylic acid	16%
Region 21.1	TransAT-PKS, NRPS, NRPS-like		
Region 23.1	NRPS, betalactone	fengycin	100%
Region 23.2	terpene		
Region 24.1	RiPP-like		
Region 25.1	NRPS	surfactin	43%
